# An exploration of emergency department presentations related to high heel footwear in Victoria, Australia, 2006–2010

**DOI:** 10.1186/1757-1146-7-4

**Published:** 2014-01-23

**Authors:** Cylie M Williams, Terry P Haines

**Affiliations:** 1 Allied Health Research Unit, Monash Health, Cheltenham, Victoria, Australia; 2 Physiotherapy Department, Monash University, Clayton, Australia; 3 Peninsula Health – Community Health, Frankston, Victoria, Australia

## Abstract

**Background:**

Many women are warned against the dangers of wearing high heel footwear however there is limited empirical evidence demonstrating an association between wearing high heel with injury. Gait laboratory testing has found a higher heel height placed the foot in a position that increases the risk of ankle sprain. Women have also been surveyed about wearing high heels and approximately half of those reported inconvenience and pain after wearing a high heel shoe. This study aims to explore emergency department presentations of injuries and the estimated costs that have been directly attributed to wearing high heeled footwear within Victoria, Australia during 2006–2010.

**Methods:**

The Victorian Emergency Minimum Dataset (VEMD) was searched for all injuries attributed to wearing high heel footwear presenting to emergency departments in Victoria Australia, between the years of 2006–2010. The VEMD produced a report detailing sex, age at presentation, month of presentation, time of day of presentation, day of presentation, location that injury occurred and type of injury for presentation. Monash Health in Victoria Australia, provided emergency department estimates for injury types to calculate an estimated cost of an acute injury related to wearing high heel footwear.

**Results:**

There were 240 injuries presenting to Victorian emergency departments directly attributed to wearing high heeled footwear. The majority of people injured were women (n = 236) and all were less than 55 years of age. More injuries presented on a Sunday (n = 83) and more in the 8 am-12 pm time bracket (n = 64). There were also more injuries presenting in the months of November, December and January (n = 80). The most commonly injured body part was the ankle (n = 123). The emergency department estimate of the cost of these injuries over this time-frame was almost $72,000 (mean of $316.72 per presentation).

**Conclusions:**

People who wear high heel footwear on weekends appear to be at higher risk for injury that leads to emergency department presentation. However, there was not a large cost associated with emergency department presentations attributable to wearing high heel footwear over a 5 year period.

## Background

The preference for high heeled footwear by women has been a concern to health professionals since the 18th century [1,2]. The 19th century saw the first warnings that wearing high heeled footwear could lead to trips and falls [3]. Since this time, women have also been warned about the possibility for long term foot change from wearing high heeled footwear including shortened calf muscles, clawed toes, sprained ankles, bunions and foot pain [4]. Medical journals have reported authors’ opinions about the problems that wearing high heeled footwear may cause to the toes, feet and legs [4-7]. Legal cases concerning women being forced to wear high heels within the working environment and their associated pain have also been waged [8]. However no direct empirical evidence supporting foot deformity or injuries in younger women has been reported.

Indirect evidence of an association between wearing high heeled footwear and harm has been generated through laboratory based studies and cross sectional surveys. For example, one laboratory based study found that wearing of heels higher than 5.08 cm significantly increased heart rate and oxygen consumption, leading the authors to recommend that only heels lower than this height should be worn under the assumption that wearing a higher heel may fatigue the wearer and therefore result in injuries [9]. One could counter-argue, however, that the greater energy expenditure when wearing high heeled footwear could be beneficial for weight loss and cardiovascular health and should therefore be encouraged [9]. A gait analysis study of frontal plane biomechanics when wearing high heeled footwear found that the higher the heel, the greater the ankle inversion moment [10]. These authors argued that wearing high heeled footwear placed the ankle at greater risk for inversion sprain injury simply due to this anatomical positioning. Further, a survey of two hundred women who regularly wore high heeled footwear, found that approximately half of the women suffered from back pain and felt limited in their every day activity when wearing a heel between 6 cm-9 cm of height [11]. While each of these studies recommended heel height reductions to prevent injury, they did not provided any direct evidence that high heel footwear lead to an increase in the rate of injury.

There is a need to further examine potential sources of evidence that may support or refute the association between wearing high heeled footwear and injuries emergency departments are one key access point to the health care system that may provide an indicator on the burden of serious injuries associated with wearing high heeled footwear. This study aims to explore emergency department presentations for injuries and the estimated emergency department costs that have been directly attributable to the wearing of high heels within Victoria, Australia during 2006–2010.

## Methods

The state of Victoria has the second highest population in Australia with the most recent census reporting a population of 5,679,000 people [12]. In the 2010–2011 period there were 1,483,159 episodes of care statewide at public emergency departments [13]. There are 38, 24 hour, public emergency departments throughout Victoria and these departments are staffed by emergency medicine physicians, specialist nurses and in many of the metro areas, allied health staff. On presentation to a Victorian emergency department, the injured patient has de-identified information recorded about the type and mechanism of injury by a triage nurse. The Victoria Emergency Minimum Dataset (VEMD) has collected and collated de-identified injurious data, together with demographic and relevant clinical information. The “type” and “mechanism of injury” fields of this data set were of interest in this study.

To conduct this study, a data request was place to the VEMD: “What injuries have presented to Victorian emergency departments between the years of 2006–2010, that were attributed to wearing high heel footwear?” The VEMD data manager conducted all searches based on the data request, where information was ambiguous or unclear; the authors discussed the reports with the data manager. As this information was collected in a de-identified manner, the VEMD does not require ethical approval to produce this type of report for research purposes.

In consultation with the authors, all recorded injury cases were searched against text narrative including “high heel”, “heel”, and “stiletto”. All cases were then manually screened for relevance. Cases were included if footwear was reported as being a mechanism for injury. Injury cases were also excluded if the mechanism of injury with footwear was due to the footwear from a second party. The VEMD produced a report detailing sex, age at presentation, month of presentation, time of day of presentation, day of presentation, location that injury occurred and type of injury for presentation.

To estimate the cost of emergency department presentations with high heeled footwear recorded as a mechanism of injury to the Victoria health system, figures were used from a 2011–2012 costing project within Monash Health hospitals in Victoria, Australia. During 2006–2010 there were a number of funding changes from the Victorian Government that did not allow for specific injury costing to be calculated, the project team providing costing estimates determined this to have minimal impact on the estimates used within this study. Based on the injury type reported from the VEMD, the injuries were grouped into four types: fractures (wrist, hand, knee, leg, foot), sprain (wrist, hand, knee, leg, ankle, foot), injury to muscle/tendon (wrist, hand, knee, leg, foot) and superficial injury to body region (excluding teeth). Cost estimates for each injury grouping were calculated based on the average cost of emergency medical staffing, emergency nursing staffing, allied health staff, medical imaging, pathology costs, pharmacy costs across each of the triage costs in 2011–2012. This costing did not include any admission or inpatient expenses that may have occurred as a result of the injury.

Chi-square analyses were undertaken to determine if the distribution of presentations across four-hour time blocks during the day was significantly different from a uniform distribution (ie. the null hypothesis is that each four hour block has the same frequency of emergency department presentations). The same approach was used to determine whether the distribution of presentations across the day of the week and the month of the year was significantly different from a uniform distribution. Post-hoc, pair-wise comparisons (individual category versus all other categories combined) were then undertaken to identify specific categories (ie. time of day, day of week, month of year) that had higher or lower frequencies of presentation than expected under a uniform distribution. Chi-square statistics were calculated using Stata IC version 11.0 [14].

## Results

**Figure 1:**
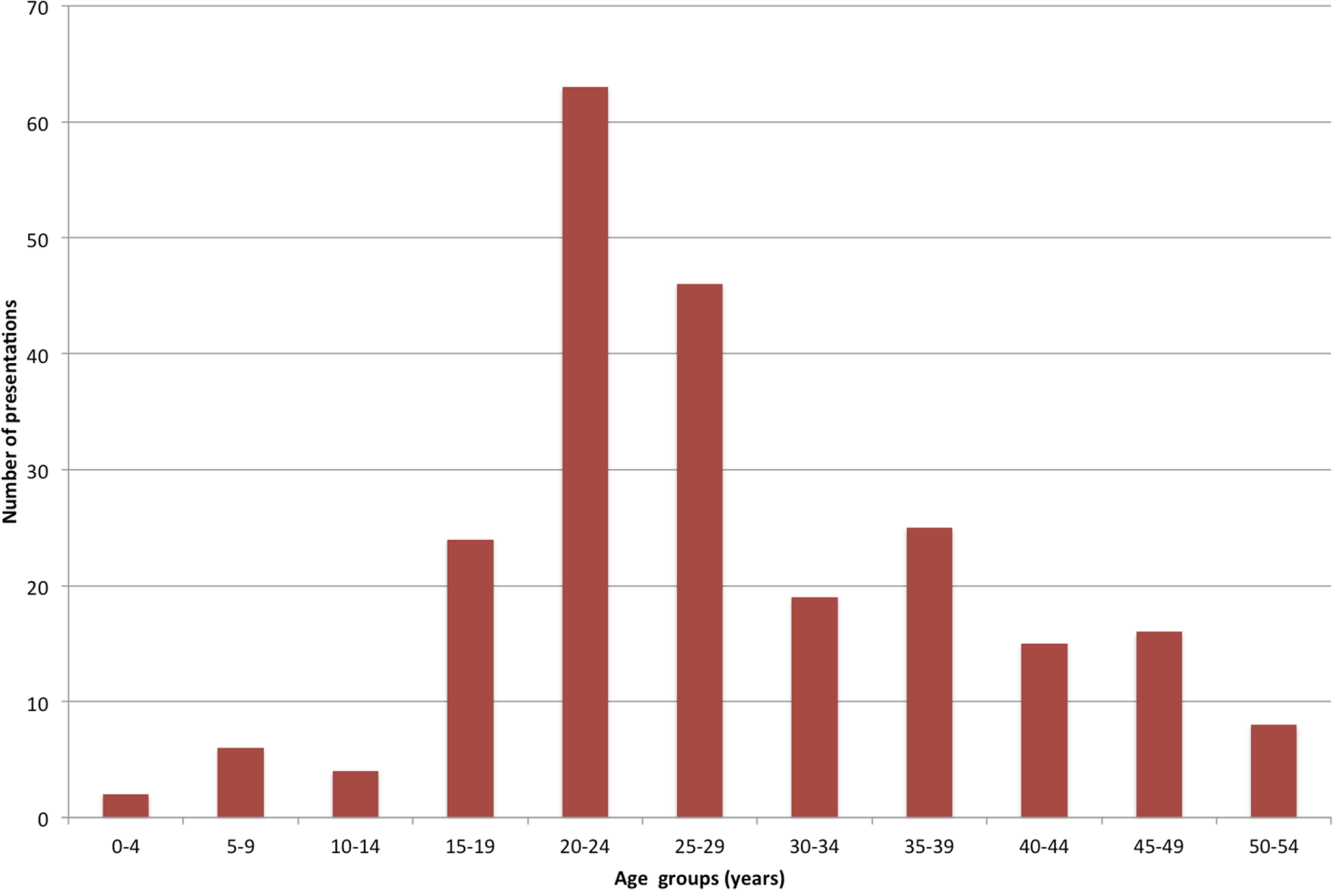
Number of presentations per age group.

There were 305 presentations to emergency departments within Victoria for injuries related to high heeled footwear. Sixty-five cases were excluded on review, as the mechanism for injury included being stepped on by a second party’s high heel shoe/stiletto (n = 49) or being assaulted with a high heel shoe/stiletto (n = 16). There were 240 emergency department presentations over the 5 year period where the patient reported the mechanism of injury being attributable to wearing high heeled footwear. Ninety-eight percent of presentations (n = 236) were female, 2% male (n = 4) and the most common age group was the 20–24 years (n = 63, 26%). There were no presentations of people over the age of 55 with an injury related to high heel footwear (Figure 1). The lower extremity was the most common body area that was injured (n = 209, 87%), with the ankle (n = 123, 51%), foot, including toes (n = 63, 26%) and the knee (n = 134, 56%) being the areas most affected (Table 1). There were also a small number of open wounds and lacerations. Falls from a height of less than 1 metre were recounted as the most common injury mechanism (n = 177, 74%). These falls occurred at varied locations including homes, places for recreation, road, street or highway (Table 2), 54 cases or 23% did not have a specified location or the location was not recorded by the triage nurse responsible for injury data collection.

**Table 1:** Location of injuries

**Body location**	**Frequency**	**Percentage**
Face, excludes eye	5	2.1
Wrist	6	2.5
Knee	15	6.3
Lower leg	5	2.1
Ankle	123	51.3
Foot, includes toes	63	26.3
Total	240	100.0

**Table 2:** Type of place where injury occurred

**Location**	**Frequency**	**Percentage**
Place for recreation	46	19.2
Home	46	19.2
Road, street or highway	44	18.3
Trade or service area	17	7.1
Other specified place	28	11.7
Unspecified place	54	22.5
Total	240	100.0

**Figure 2:**
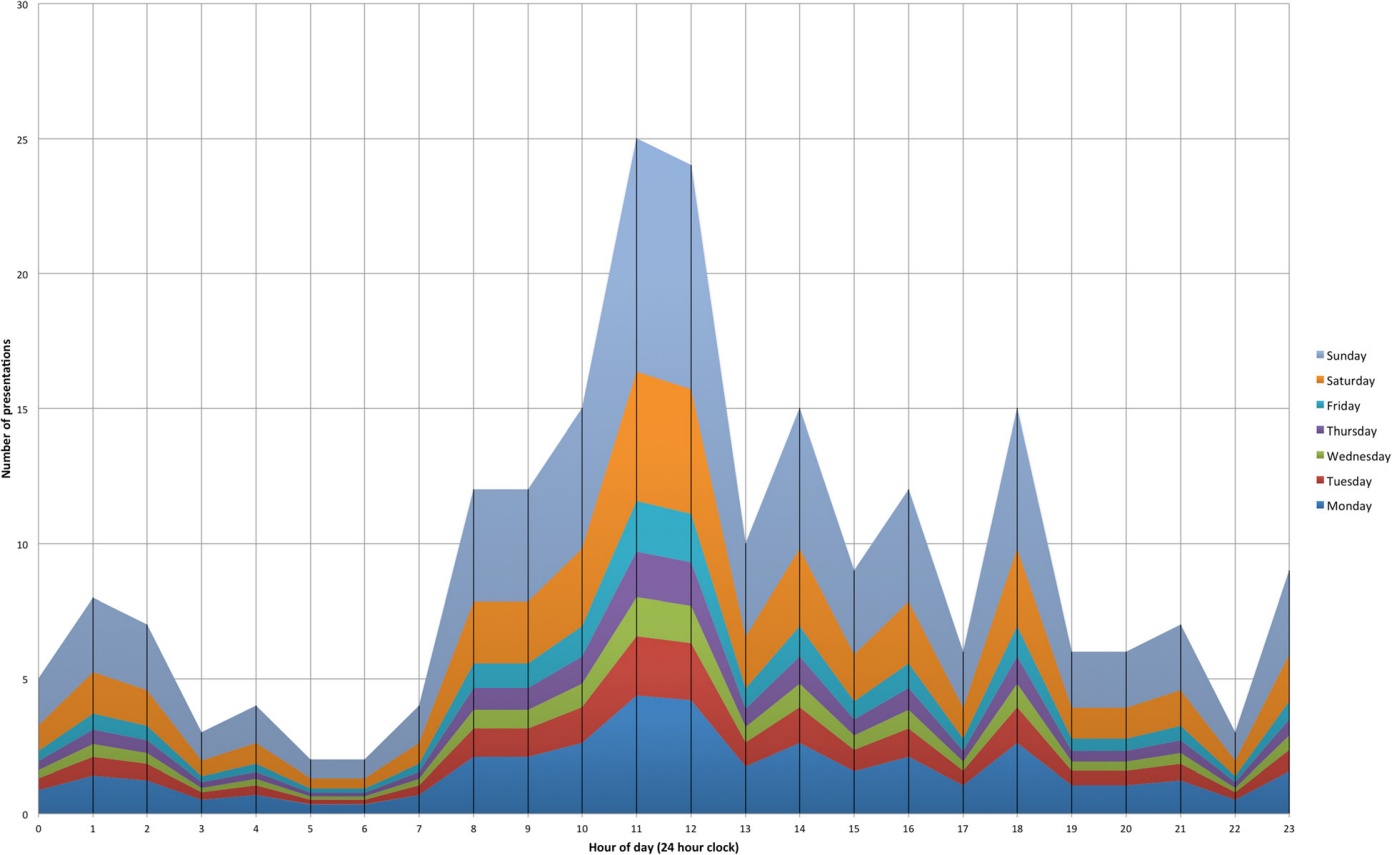
Number of presentations during the days and hours of the week.

The distribution of presentations across the time of day, day of week and month of year were not uniform (time of day: *X*^2^ = 58.07, df = 5, p < 0.001 day of week: *X*^2^ =109.46, df = 6, p < 0.001, month of year: *X*^2^ = 25.2, df = 11, p = 0.009). Weekends had a higher frequency of presentation than was expected (Saturday *versus* all other days combined: *X*^2^ = 4.709, df = 6, p = 0.58 Sunday *versus* all other days combined: *X*^2^ = 80.779, df = 6, p < 0.001, Saturday and Sunday *versus* all other days combined: *X*^2^ = 94.547, df = 6, p < 0.001). Sunday had the highest frequency of presentations overall (n = 83) (Figure 2). Time of day influenced the presentation of injuries related to high heel use when considering 4 hour blocks across the day. Presentations were most common in the 8 am-12 pm time bracket (n = 64, 8 am-12 pm *versus* all other time categories combined: *X*^2^ = 24.078, df = 5, p = 0.002) and least common in the 4 am-8 am time bracket (n = 12, 4 am-8 am *versus* all other time categories combined: *X*^2^ = 20.113, df = 5, p = 0.001). A further post-hoc comparison between business hours (Monday-Friday, 8 am-6 pm) against all other hours, did not identify a significant difference in the presentation frequency (*X*^2^ = 0.013, df = 1, p = 0.91) however when restricted just to Monday-Friday data, business hours were significantly higher in their frequency of presentation compared to non-business hours on those week days. (*X*^2^ = 19.47, df = 1, p < 0.001). Time of year was examined in a post-hoc analysis where a three month time period of November through to January was grouped and compared to the remaining three month period groups. This analysis indicated that emergency department presentations did vary significantly over the time of year with the greatest number of injuries over the November to January time period (n = 80, November to January *versus* n = 57, Febuary to April, n = 56, May to July, n = 47, August to October: *X*^2^ = 9.900, df = 3, p = 0.0194).

The estimated total costing of high heel related injuries to Victorian Emergency departments for the period of 2006–2010 was $71,579.62 (Table 3) based on the injury estimates provided.

**Table 3:** Costing estimate for injury based on Monash Health emergency department estimates

**Injury type**	**Average cost per attendance**	**Frequency**	**Total estimated costs**
Fractures (inc Wrist, Hand, Leg, foot )	$694.48	53	$36,807.44
Sprain (Inc wrist/hand/ankle/knee)	$181.55	134	$24,327.70
Injury to muscle/tendon (Inc wrist/hand/ankle/knee)	$239.24	16	$3,827.84
Superficial injury	$287.68	23	$6,616.64
*Total for all injuries (n = 226)			$71,579.62

*14 injuries unaccounted for due to missing data.

## Discussion

Sensationalist media reports on the dangers of high heel wearing have appeared frequently in newspapers, online and television media, however there has been very little evidence reporting a direct relationship between high heel footwear and foot injury. While footwear has been directly related to injuries in older women [15], this analysis of high heel footwear injuries has been the first study to examine the relationship between high heel footwear and injuries in younger women and to provide an estimated hospital cost for acute presentation.

The distribution of presentations over time of day, day of week and month of year provides impetus to speculate as to what may have caused these patients to present at these times. Although not measured in this study, it was anticipated by the authors that high heeled footwear would not be more likely worn on Sundays or the 8 am till 12 pm time slot than other days or time slots. It could be hypothesized that the injury took place on the previous night and that presentation to the emergency department may have been delayed due to misperception of the severity of the injury or initial attempts to self manage eventually failed. The need for another person to transport the patient to the emergency department (if not being transported by ambulance) may also have led to the greater proportion of morning presentations.

The presentation of injuries during the warmer months and around culturally significant events was an interesting finding. Melbourne’s “Spring Racing Carnival” horse racing events are held throughout November in Victoria and have a strong focus on fashion. At this time also there are generally larger numbers of people at indoor and outdoor recreation venues often where there is alcohol consumption and uneven flooring surfaces or terrain. Similarly, seasonal festivities during December and January may also be the precipitant for an increase in women wearing high heel footwear, leading to increased serious injuries. It would be interesting to see if other Australian states have a similar distribution pattern of injuries related to high heel footwear or if this finding was particular to Victoria.

The majority of injuries associated with high heel footwear were ankle sprains. Ankle sprains are a common injury in sport and there has been no evidence supporting a reduction of quality of life related to this injury. Ankle sprains have been reported to take up to six years to fully recover [16,17] and people generally required allied health assisted rehabilitation, bracing or splinting. There has also been long term swelling of the ankle, pain at the foot and ankle and instability around the ankle joint, associated with ankle sprains [16]. No health economic data has been found in order to provide an estimate for the assessment and initial treatment of acute ankle sprains other than the estimate used within this study. There were also no estimates found within the literature for the longer term chronic ankle sprain rehabilitation, nor for time off work, medications and any supportive aids (eg. ankle braces) that may be required. Therefore, the overall burden of injuries arising from use of high heeled footwear is likely to be higher than that presented in this paper.

Our study was limited in its ability to capture the overall frequency of injuries related to high heeled footwear use. Reliance on hospital emergency department presentations means that we likely only captured the more severe end of the spectrum of injuries arising. The frequency of injuries observed would likely be far greater if presentations to general practitioners and allied health professionals were available. Another limitation of this study was that it is possible that triage staff within each emergency department did not enquire of or record the role of high heeled footwear in the mechanism of injury field in the patient notes. It is also possible that the injured patient did not mention footwear as the mechanism for injury, or if there was also alcohol involved, intoxication may have been recorded as the primary cause of injury rather than the combination of high heel footwear (which does not have its own field) and intoxication (which has its own field). The calculation of emergency department costing estimates were based on funding calculations after the injuries data set time frame and therefore the estimates used would have been slightly inflated. Despite this limitation it is presumed these estimates may be a conservative measure of health cost for the acute injury related to high heel footwear due to the potential for non-recording of high heel related injuries during triage. Lastly, there is a limitation in how the high heel footwear may have been categorized by triage nursing staff while recording footwear type. “High heel” or “stilettos” as a category of high heel footwear may be applied to any footwear that has a small stiletto heel of approximately 2 cm through to the increasingly popular extreme heels, the shoe with a platform at the front of the shoe which increases the heel height and gives a potential overall heel height of up to 15 or 20 cm. These variables make it challenging to determine the real impact of the height of the heel on the footwear to the injury.

## Conclusions

The foot health impact of fashion is not well understood in regards to the choices that younger women make about footwear. High heel footwear has been directly related to lower limb injuries in this study and it is proposed that the numbers reported within the VEMD are potentially under representative of these injury types. Within a 5 year period, the acute high heel related injuries are estimated to cost the Victorian health system almost $72,000 however this burden is potentially an under estimate. To better understand injuries attributable to high heel use, future research should include the private sector of medical and allied health clinicians, together with wearers of high heel shoes as data collection sources. Weekend wear of high heel footwear appears to place women at greater risk of lower limb injury. While women continue to make these shoe choices, more work is needed to better to understand why these choices are made and how to minimize any risks associated with their wear.

## Competing interests

The authors have no competing interests to declare.

## Authors’ contributions

Both authors (CMW & TPH) equally designed the study, analyzed the data and drafted the manuscript. Both authors read and approved the final manuscript.
